# IgG Isotypes Targeting a Recombinant Chimeric Protein of *Trypanosoma cruzi* in Different Clinical Presentations of Chronic Chagas Disease

**DOI:** 10.4269/ajtmh.23-0652

**Published:** 2024-02-27

**Authors:** Isabela Machado Serrano, Gilmar Ribeiro, Ronnei Silva Santos, Jaqueline Silva Cruz, Fernanda Cardoso Lanza, Emily Ferreira dos Santos, Márcio Cerqueira de Almeida, Jorgana Fernanda de Souza Soares, Alejandro Ostermayer Luquetti, Paola Alejandra Fiorani Celedon, Nilson Ivo Tonin Zanchin, Fred Luciano Neves Santos, Mitermayer Galvão dos Reis

**Affiliations:** ^1^ Laboratory of Pathology and Molecular Biology, Gonçalo Moniz Institute, Oswaldo Cruz Foundation–Bahia, Salvador, Brazil;; ^2^ Advanced Health Public Laboratory, Gonçalo Moniz Institute, Oswaldo Cruz Foundation–Bahia, Salvador, Brazil;; ^3^ Integrated Translational Program in Chagas Disease, Oswaldo Cruz Foundation, Rio de Janeiro, Brazil;; ^4^ Faculty of Medicine, Federal University of Bahia, Salvador, Brazil;; ^5^ Chagas Disease Study Center, University Hospital, Federal University of Goiás, Goiânia, Brazil;; ^6^ Laboratory of Molecular and Systems Biology of Trypanosomatids, Carlos Chagas Institute, Oswaldo Cruz Foundation–Paraná, Curitiba, Brazil;; ^7^ Laboratory of Structural Biology and Protein Engineering Laboratory, Carlos Chagas Institute, Oswaldo Cruz Foundation–Paraná, Curitiba, Brazil;; ^8^ Department of Epidemiology of Microbial Diseases, Yale School of Public Health, Yale University, New Haven, Connecticut

## Abstract

Chagas disease (CD) is caused by the protozoan *Trypanosoma cruzi*, which leads to a spectrum of clinical presentations that range from asymptomatic to severe cardiac involvement. The host immune response plays a pivotal role in disease progression. Ig isotypes may contribute to disease pathogenesis. Investigating these components can provide insights into the immunopathogenic mechanisms underlying CD. This cross-sectional study aims to establish a correlation between the Ig profile of individuals infected with *T. cruzi* with the clinical forms of chronic CD. Serum samples were collected from partner institutions in different states of Brazil. Individuals diagnosed with chronic CD were categorized based on the clinical form of the disease. The indirect ELISA method using the recombinant chimeric Molecular Biology Institute of Paraná membrane protein 8.4 as the antigen was used to determine the Ig profile, including total IgG, IgG1, IgG2, IgG3, and IgG4. Ninety-seven serum samples from patients classified as negative (NEG, *n =* 38), indeterminate (IND, *n =* 24), mild cardiac (MC, *n =* 20), and severe cardiac (SC, *n =* 15) forms were analyzed. IgG1 exhibited greater levels compared with the other isotypes, showing a significant difference between the MC and IND groups. IgG3 levels were greater in individuals from the MC group compared with the SC group. IgG1 and IgG3 isotypes can serve as biomarkers to evaluate the progression of CD because they exhibit variations across clinical groups. Additional longitudinal studies are necessary to explore the relationship between antibody kinetics and the development of tissue damage.

## INTRODUCTION

Chagas disease (CD) is a vector-borne, neglected tropical condition caused by a hemoflagellated protozoan parasite *Trypanosoma cruzi*. This parasite imposes a significant health burden in 21 Latin American countries, with approximately 6–7 million cases and 7,500 deaths annually.^1^^,^^2^ An estimated 75 million people are at risk of contracting the disease worldwide.^2^ In endemic regions, *T. cruzi* is transmitted primarily through contact with feces or urine of infected bloodsucking triatomine insects, which carry the parasite in their intestines. Other modes of transmission include ingestion of contaminated food and beverages, organ transplantation, mother-to-child transmission, blood transfusion, and, less commonly, laboratory accidents. Human migration has played a role in the global expansion of CD during the past few decades, reaching nonendemic countries in Europe, North America, Asia, and Oceania.^3^^–^^5^

Chagas disease is characterized by two distinct phases. The initial phase, which occurs shortly after infection and lasts up to 2 months, is typically asymptomatic but is accompanied by high parasitemia. Symptoms, when present, are nonspecific and manifest as a self-limiting febrile illness.^6^ Following the acute phase, infected individuals enter in the chronic phase, which may be in different clinical forms: a long-lasting asymptomatic form known as the indeterminate form. In this form, which can last for years or even decades, there are no apparent clinical manifestations. However, approximately 20–30% of affected individuals eventually progress to a symptomatic form, characterized by severe complications in the heart (cardiac form) or digestive tract (megaesophagus or megacolon), or both.^6^ The diverse clinical manifestations and varying severity of symptoms observed in CD have been attributed by some researchers to differences in the host’s immune response and the differential expression of proteins by different strains of *T. cruzi*.^7^^,^^8^

The immune response to *T. cruzi* infection is intricate, and involves both innate and adaptive immunity.^9^ The pathogen has developed multiple mechanisms to evade the host immune response.^10^^–^^12^ One such mechanism is the induction of diverse Ig isotypes, which can either contribute to host resistance against infection or promote tissue damage by enhancing inflammatory responses.^13^ Studies investigating isotypes in individuals with CD and cardiac involvement have produced conflicting findings. Some studies^14^^,^^15^ have reported greater IgG1 titers in severe cardiac forms, whereas others^7^^,^^16^^,^^17^ have found elevated IgG2 titers. These discrepancies can be attributed to factors such as the genetic profiles of the populations studied, geographic location, variations in serological methods, parasite strain, and antigen preparation.^18^ Various antigen preparations have been used, including epimastigote extracts from Tulahuen and Y strains,^15^^–^^17^ cytosolic acidic antigen fractions,^14^ and recombinant cytoplasmic and flagellar antigens.^7^ To reduce this variability, chimeric recombinant antigens have emerged as a strategy, because they can be produced consistently in large quantities using bioreactors. Chimeric molecules incorporate conserved and repetitive amino acid sequences from different parasite proteins, which enables the detection of IgG isotypes even at low expression levels.^19^

In light of this scenario, our team synthesized and purified four recombinant chimeric *T. cruzi* antigens (Molecular Biology Institute of Paraná [IBMP-8.1, IBMP-8.2, IBMP-8.3, and IBMP-8.4) and investigated their diagnostic potential for detecting anti-*T. cruzi* IgG in humans^19^^–^^28^ and dogs.^29^ The antigen IBMP-8.4 exhibited superior sensitivity, specificity, and accuracy across endemic and nonendemic regions, including Spain,^22^ Brazil,^20^^,^^21^ and other Latin American countries.^23^ Based on these findings and the larger repertoire of epitopes present in its protein composition,^19^^,^^30^^,^^31^ we selected the IBMP-8.4 molecule for this study. Because there is a dearth of studies examining the involvement of IgG isotypes in the presence of this type of antigen among characterized clinical patients, our aim was to examine the profile of Ig isotypes across various clinical forms of chronic CD, with the potential to identify immunological markers associated with the progression of cardiac disease.

## MATERIALS AND METHODS

### Serum samples.

**Figure 1.**
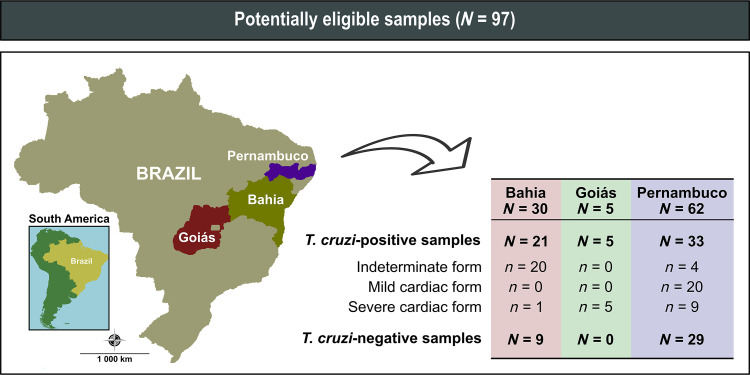
Geographic location of sample collection sites in this study. Source base layer and credit base layer: https://data.humdata.org/ published under creative commons attribution for intergovernmental organizations and https://data.humdata.org/dataset/geoboundaries-admin-boundaries-for-brazil. Reprinted with permission.

Ninety-seven sera were obtained from *T. cruzi*–positive (*n =* 59) and *T. cruzi*–negative individuals (*n =* 38) (Figure 1). Samples from *T. cruzi*–positive individuals were confirmed by serological tests (indirect hemagglutination assay, indirect immunofluorescence test, ELISA, and chemiluminescence) and classified by partner institutions into different clinical stages of infection: indeterminate (IND) form of CD (*n =* 24), mild cardiac (MC) form of CD (*n =* 20), and severe cardiac (SC) form of CD (*n =* 15). Following the “SBC Guideline on the Diagnosis and Treatment of Patients with Cardiomyopathy of Chagas Disease,”^32^ the IND form (stage A) exhibited no symptoms or signs, with normal findings in electrocardiographs, chest radiographs, and gastrointestinal examinations. The MC form (stage B1) presented electrocardiographic changes (conduction abnormalities or arrhythmias) and mild echocardiographic changes (regional contractility abnormalities) without ventricular dysfunction. The SC form (stage C) was characterized by electrocardiographic and echocardiographic changes, along with ventricular dysfunction. Detailed clinical and sociodemographic information, including age, gender, and geographic origin, was recorded during the blood collection process. *Trypanosoma cruzi*–negative sera were sourced from volunteer blood donors at the Foundation for Hematology and Hemotherapy of Bahia and Foundation for Hematology and Hemotherapy of Pernambuco, and were confirmed as negative through chemiluminescence assays. In addition, these samples tested negative for HIV-1/2, human T-lymphotrophic virus 1/2, and syphilis, hepatitis B, and hepatitis C viruses.

### Evaluation of total IgG and IgG isotypes against *T. cruzi* IBMP-8.4 by indirect ELISA.

We performed the detection of IgG anti-*T. cruzi* in duplicate using an indirect ELISA method with a recombinant chimeric *T. cruzi* protein (IBMP-8.4) as the antigen, according to a previously described protocol,^19^ with some modifications. Checkerboard titration was conducted to determine optimal dilutions of antigen coating, antibody-peroxidase conjugate (horseradish peroxidase [HRP]), and serum concentrations for detecting of IgG isotypes (anti-IgG1, -IgG2, -IgG3, and -IgG4). The selected conditions were based on the greatest signal-to-noise ratio (SNR), representing the ratio of mean optical density (OD) values between positive and negative samples. Flat-bottom, high-binding, transparent Maxisorp 96-well microplates (Nunc, Rochester, NY) were used for coating with the IBMP-8.4 antigen (12.5, 25, and 50 ng) in carbonate-bicarbonate buffer (50 mM; pH, 9.6). After blocking with Well Champion reagent (lot 130703; Kem-En-Tec Diagnostics A/S, Taastrup, Denmark), 100 µL of each serum sample (diluted 1:100 and 1:200 in phosphate-buffered saline; pH, 7.4) was added to the designated well, followed by a 60-minute incubation at 37°C. Subsequently, the microplates were washed with phosphate-buffered saline–0.05% Tween 20, and 100 µL of HRP-conjugated mouse anti-human IgG1 (A-10648; Thermo Scientific, Rockford, IL), IgG2 (MH1722, Thermo Scientific), IgG3 (MH1732, Thermo Scientific), and IgG4 (A-10654, Thermo Scientific) diluted at 1:500, 1:1,000, 1:2,000, 1:10,000, 1:20,000, and 1:40,000 ratios in phosphate-buffered saline were added to the respective wells. The microplates were then incubated at 37°C for 30 minutes. After another washing cycle, 100 µL of 3,3′,5,5′-Tetramethylbenzidine substrate (lot 111011; Kem-En-Tec, Taastrup, Denmark) was added to each well, and the microplates were incubated in the dark at room temperature for 10 minutes. The colorimetric reactions were stopped by adding 50 µL of 0.3 M sulfuric acid to each well, and the OD was measured using a 450-nm filter (Sunrise,^TM^ Tecan, Switzerland).

## STATISTICAL ANALYSES

Data analysis was conducted using scatterplot software (GraphPad Prism Software, version 10.0.0, San Diego, CA). Descriptive statistics included frequencies for qualitative variables (gender and geographic origin) and median ± interquartile range (IQR) (or arithmetic mean ± SD for ELISA standardization) for quantitative variables (age, OD, and reactivity indices [RIs]). Normality of the data sets was assessed using the Shapiro-Wilk test. In cases in which variance homogeneity assumptions were not met, the Mann-Whitney test was used. All analyses were two tailed, and statistical significance was defined as *P* <0.05. To establish relevant cutoff values for the IBMP-8.4 antigen, *10 T. cruzi*–positive and *10 T. cruzi*–negative samples were analyzed alongside each microtiter plate. These samples were characterized previously as positive or negative using two serological tests according to international guidelines.^33^^,^^34^ Cutoff point analysis was performed to identify the optimal OD value that distinguishes negative from positive samples. The threshold value was defined by measuring the largest distance from the diagonal line formed by the end points of the receiver–operating characteristic curve: [Sensitivity × (1 – Specificity)]. All results are expressed by plotting the values as an index format, representing the ratio between a given sample’s OD and the threshold OD pertaining to each microplate. This index is referred to as a reactivity index, and all results ≥1.00 were considered positive.

## RESULTS

Optimal assay conditions were determined by maximizing the SNR between the mean OD values of positive and negative samples. As a result, dilution rates of 1:40,000 and 1:500 were established for HRP-labeled anti-total IgG and HRP-labeled anti-IgG isotypes, respectively. The greatest SNR values were achieved with a serum dilution of 1:100 and a coating of 25 ng of IBMP-8.4 on the plates. With these optimized conditions, we analyzed the Ig isotype profiles in different clinical forms of chronic CD using sera from 97 individuals (Figure 1; negative, *n =* 38; IND form, *n =* 24; MC form, *n =* 20; SC form, *n =* 15) who had been tested previously for *T. cruzi* infection. Individuals who were negative for *T. cruzi* had a mean age of 33.5 years (IQR, 28–40.3 years) and a female-to-male ratio of 1:2.8. In contrast, *T. cruzi*-positive individuals had a mean age of 47 years (IQR, 36–55 years) and a female-to-male ratio of 0.8:1 (*P* <000.1). Individuals who were positive for *T. cruzi* with the IND form had a mean age of 36.5 years (IQR, 33–41 years), with a female-to-male ratio of 0.6:1. Among those *T. cruzi*–positive individuals classified exclusively with the MC form, the mean age was 53.5 years (IQR, 48–61.75 years) with an equal female-to-male ratio of 1:1, whereas infected individuals classified as having the SC form had a mean age of 53 years (IQR, 47–61 years) with a female-to-male ratio of 0.88:1. Figure 1 illustrates that the majority of patients were from Pernambuco (63.9%), followed by Bahia (30.9%), and Goiás (5.2%). Figure 1 illustrates the geographic origin of both *T. cruzi*–positive and –negative individuals included in our study.

**Figure 2.**
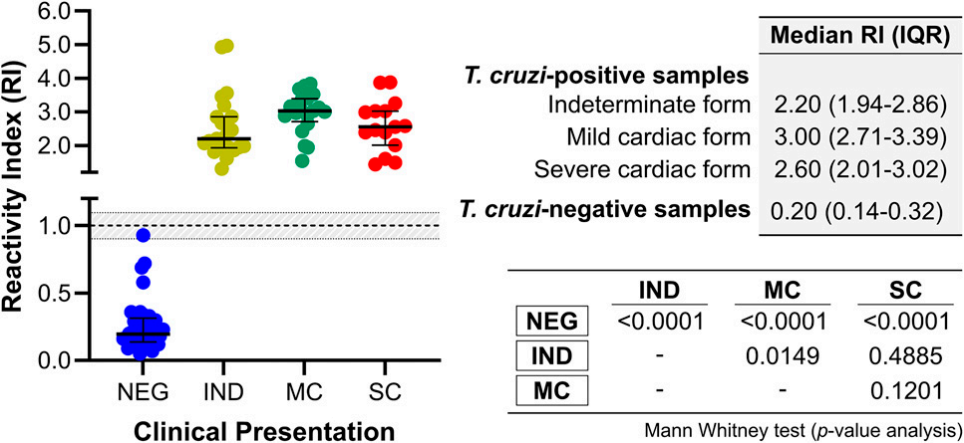
Graphical analysis of the reactivity index (RI) obtained with serum samples of *Trypanosoma cruzi*–positive and *T. cruzi*–negative (NEG) samples against total IgG against the *T. cruzi* IBMP-8.5 antigen. The cutoff value is RI = 1.0. The shaded area represents the gray zone (RI, 1.0 ± 0.10). IND = samples from *T. cruzi*–positive individuals with indeterminate form; IQR = interquartile range; MC = samples from *T. cruzi*–positive individuals with mild cardiac form; SC = samples from *T. cruzi*–positive individuals with severe cardiac form.

We assessed initially the median RIs measuring total IgG for samples from *T. cruzi*–negative individuals and different clinical presentations of *T. cruzi*–positive individuals (individual data points are provided in Supplemental Table 1). As depicted in Figure 2, the highest median RI was observed in individuals with the MC form (RI, 3.00; IQR, 2.71–3.39), followed by the SC form (RI, 2.60; IQR, 2.01–3.02), the IND form (RI, 2.20; IQR, 1.94–2.86), and, last, the panel of *T. cruzi*–negative samples (RI, 0.20; IQR, 0.14–0.32). Significant differences were found between the median RIs of *T. cruzi*–negative samples and all clinical presentations of *T. cruzi*–positive samples. A significant difference was observed in the sera from *T. cruzi*–positive individuals, specifically between samples from individuals with the IND form and those with the MC form.

**Figure 3.**
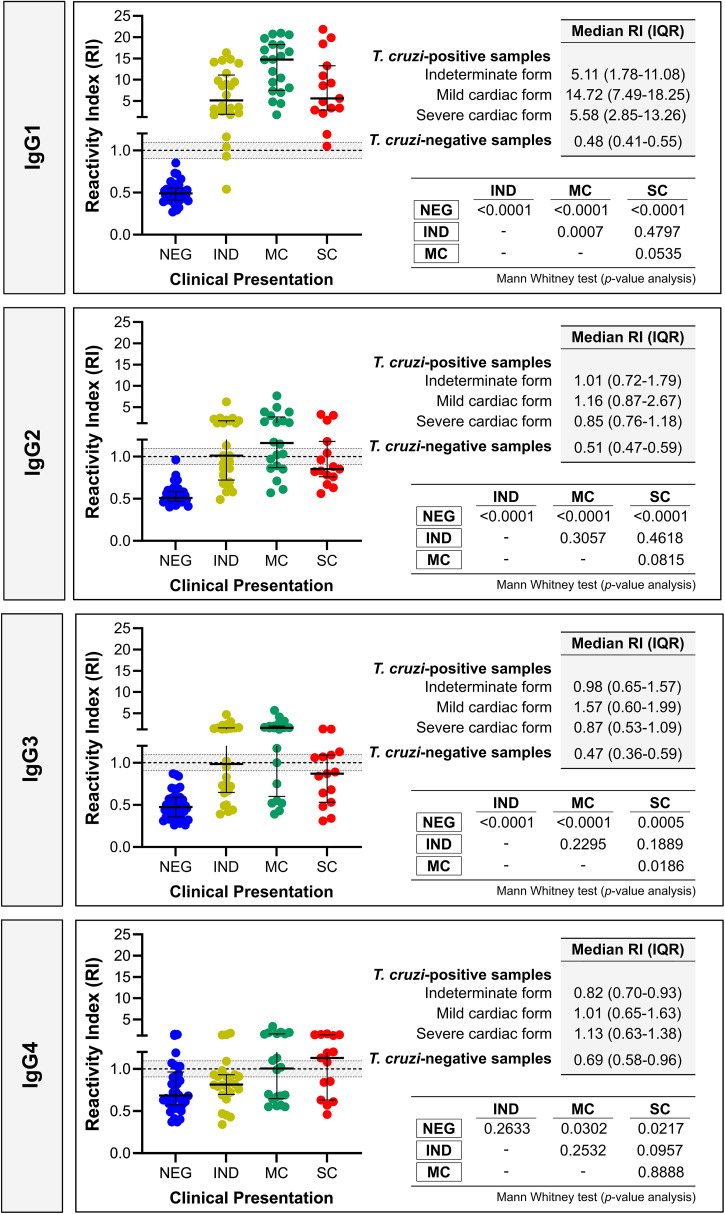
Graphical analysis of the reactivity index (RI) obtained with serum samples of *Trypanosoma cruzi*–positive and *T. cruzi*–negative (NEG) samples against IgG isotypes against the *T. cruzi* IBMP-8.4 antigen. The cutoff value is RI = 1.0. The shaded area represents the gray zone (RI, 1.0 ± 0.10). IND = samples from *T. cruzi*–positive individuals with indeterminate form; IQR = interquartile range; MC = samples from *T. cruzi*–positive individuals with mild cardiac form; SC = samples from *T. cruzi*–positive individuals with severe cardiac form.

Figure 3 illustrates the median RIs for IgG isotypes based on the clinical presentation of *T. cruzi*–positive individuals (individual data points are provided in Supplemental Table 1). Among the four IgG isotypes, IgG1 exhibited the highest median RIs, followed by IgG2, IgG3, and IgG4 across all clinical presentations. Consistent with the total IgG analysis, individuals with the MC form showed the highest median RIs, particularly for IgG1 (RI, 14.72; IQR, 7.49–18.25), IgG2 (RI, 1.16; IQR, 0.87–2.67), and IgG3 (RI, 1.57; IQR, 0.60–1.99). Significant differences were observed for IgG1 between individuals with the IND and MC forms. Although samples from individuals with the MC form had the highest median RI for IgG2, no differences were observed among other clinical presentations. In the case of IgG3, the median RI signal for samples from individuals with the MC form was significantly higher than that of samples from individuals with the SC form. There were no differences among the three clinical manifestations of *T. cruzi*–positive individuals for the IgG4 isotype. Notably, significant differences were found between samples from *T. cruzi*–negative individuals and those from *T. cruzi*–positive individuals, regardless of clinical presentation, except for IgG4, which exhibited no differences specifically in *T. cruzi*–positive individuals with the IND form.

## DISCUSSION

Despite the discovery of CD more than 114 years ago, significant questions remain unanswered, particularly regarding the identification of a biological marker to assess disease progression from asymptomatic to organ-specific symptomatic manifestations. Research groups worldwide have made numerous attempts to find biomarkers for this purpose. Some investigations have focused on specific targets for anti-*T. cruzi* antibodies,^35^^–^^37^ production of cytokines and chemokines,^38^^–^^40^ and correlations between seropositivity and electrocardiographic alterations^41^; or have explored alternative pathways such as immunoproteomics,^42^ Th17 cells,^43^ or cardiac injury markers.^44^ However, a consensus on a definitive marker has not yet been reached. In our study, we investigated the efficacy of a recombinant chimeric *T. cruzi* antigen used widely as a diagnostic tool to detect anti-*T. cruzi* antibody levels in different clinical presentations of CD, including the IND, MC, and SC forms. Notably, we observed a significant difference in RIs between samples from *T. cruzi*–positive individuals with the IND form and those with the MC form when testing total IgG or IgG1 isotype anti-*T. cruzi* with IBMP-8.4.

IgG1 is the predominant isotype of antibodies in the human body, constituting approximately 70% of total circulating antibodies.^45^ Our study revealed increased levels of IgG1 in the sera of patients with MC disease. This finding aligns with previous reports highlighting the high titers of IgG1 and its involvement in proinflammatory mechanisms, including lytic activity and complement system activation. This suggests the potential for using IgG1 levels to differentiate between *T. cruzi*–positive and –negative individuals.^7^^,^^46^ However, the precise inflammatory role of these antibodies in cardiac fibers remains complex, and there is limited information regarding the progression of cardiac damage from mild to severe or the maintenance of cardiac integrity.^47^^,^^48^ Notably, a retrospective longitudinal cohort study^15^ demonstrated a correlation between elevated IgG1 levels and left ventricular ejection fraction dysfunction. The authors suggested a potential association with cardiomyopathies, as the kinetics of anti-*T. cruzi* antibodies indicate a progression toward SC damage, which could have prognostic implications for cardiac diseases. It is important to note that our study used clinically precharacterized samples without patient follow-up, thus limiting our ability to assess disease progression.

Regarding the anti-*T. cruzi* IgG3 isotype, we found a significant difference in RIs between the MC and SC forms. This finding could suggest a potential role in cardiac injury; a regulatory function could potentially mitigate the severity of tissue damage. Indeed, these regulatory mechanisms may be influenced by factors produced and released by the parasite. In addition, the progression of SC lesions might involve the cytotoxic profile of TCD8+ cells,^49^ although this aspect was not explored in our study.

As for the IgG2 and IgG4 isotypes, we did not observe any significant differences between the clinical groups. However, previous studies have indicated a potential association between IgG2 and the cardiac form.^7^ In fact, high maternal IgG1 and IgG2 levels have been demonstrated to be highly predictive of congenital transmission of CD, highlighting their importance in this context.^50^ In our study, we found low RIs for IgG4, without any significant differences among the clinical groups. Conversely, elevated levels of IgG4 have been reported in individuals with the digestive form of CD.^51^^–^^53^

Our findings reveal elevated levels of IgG1 and IgG3 isotypes in individuals with the cardiac form of CD, suggesting their potential involvement in the antibody-mediated immune response within the myocardium and their potential association with disease severity. Remarkably, a previous study^54^ has already recognized these isotypes as markers for both pre- and end-stage heart failure. Furthermore, investigations^55^^,^^56^ focusing on patients before and after benznidazole treatment have demonstrated that monitoring IgG1 and IgG3 levels in individuals with cardiac CD may indicate the progression of cardiomyopathy and may serve as valuable prognostic biomarkers. Consequently, comprehensive studies encompassing additional antibodies, cytokines, chemokines, and cell markers in the presence and absence of the parasite within cardiac fibers could provide additional insights into the interaction between *T. cruzi* and the immune system, particularly within representative and comparable clinical groups. It is important to highlight that the levels of anti-*T. cruzi* IgG3 and IgG1 isotypes did not demonstrate significant differences when comparing samples from patients with the MC and SC forms. However, the low *P*-value suggests that an increase in the sample size in future studies might be sufficient for these isotypes to distinguish antibody levels between these groups.

A significant limitation of our study is its cross-sectional design, which precludes patient follow-up. Considering the slow progression of cardiac injury, a long-term prospective cohort study is required. Despite this constraint, our findings highlight the promising capacity of the IBMP-8.4 molecule in distinguishing between clinical groups based on the form of CD. Future investigations should consider incorporating other IBMP proteins (IBMP-8.1, IBMP-8.2, and IBMP-8.3).^19^

This study aimed to characterize the IgG isotypes present in different clinical forms of chronic CD using the IBMP-8.4 molecule as an antigen. Our findings demonstrate increased reactivity of IgG1 compared with other isotypes, particularly in individuals with the MC form in comparison to those with the IND form. Moreover, higher levels of IgG3 were elevated in individuals with the MC form in contrast to those with the SC form. These results highlight the necessity for further investigation into the relationship between antibody levels, tissue damage, and clinical progression to identify prognostic biomarkers for CD.

## Supplemental Materials

10.4269/ajtmh.23-0652 Supplemental Materials
