# Supplementary material for: IgG Isotypes Targeting a Recombinant Chimeric Protein of Trypanosoma cruzi in Different Clinical Presentations of Chronic Chagas Disease
Source: Am J Trop Med Hyg. 2024 Feb 27;110(4):669–76. doi: 10.4269/ajtmh.23-0652 (PMC10993828; doi:10.4269/ajtmh.23-0652)
Supplement: Supplemental Materials [file tpmd230652.SD1.pdf]

Supplemental Table 1

Reactivity index values for total IgG and IgG isotypes using IBMP-8.4 indirect ELISA

| Sample | Clinical presentation | IBMP-8.4 - ELISA (reactivity index) | | | | |
|---|---|---|---|---|---|---|
| | | Total IgG | IgG1 | IgG2 | IgG3 | IgG4 |
| *Trypanosoma cruzi* -negative 1 | Negative | 0.19 | 0.60 | 0.46 | 0.51 | 0.58 |
| *Trypanosoma cruzi* -negative 2 | Negative | 0.14 | 0.32 | 0.54 | 0.42 | 0.92 |
| *Trypanosoma cruzi* -negative 3 | Negative | 0.13 | 0.51 | 0.47 | 0.50 | 0.80 |
| *Trypanosoma cruzi* -negative 4 | Negative | 0.19 | 0.41 | 0.61 | 0.36 | 0.41 |
| *Trypanosoma cruzi* -negative 5 | Negative | 0.20 | 0.43 | 0.41 | 0.87 | 0.54 |
| *Trypanosoma cruzi* -negative 6 | Negative | 0.16 | 0.41 | 0.56 | 0.86 | 0.68 |
| *Trypanosoma cruzi* -negative 7 | Negative | 0.21 | 0.41 | 0.47 | 0.57 | 0.86 |
| *Trypanosoma cruzi* -negative 8 | Negative | 0.25 | 0.52 | 0.49 | 0.84 | 0.63 |
| *Trypanosoma cruzi* -negative 9 | Negative | 0.36 | 0.47 | 0.57 | 0.70 | 1.24 |
| *Trypanosoma cruzi* -negative 10 | Negative | 0.29 | 0.41 | 0.52 | 0.33 | 0.37 |
| *Trypanosoma cruzi* -negative 11 | Negative | 0.18 | 0.36 | 0.47 | 0.26 | 1.49 |
| *Trypanosoma cruzi* -negative 12 | Negative | 0.10 | 0.52 | 0.48 | 0.44 | 0.94 |
| *Trypanosoma cruzi* -negative 13 | Negative | 0.09 | 0.53 | 0.72 | 0.41 | 1.19 |
| *Trypanosoma cruzi* -negative 14 | Negative | 0.12 | 0.52 | 0.51 | 0.44 | 1.03 |
| *Trypanosoma cruzi* -negative 15 | Negative | 0.33 | 0.60 | 0.47 | 0.59 | 0.72 |
| *Trypanosoma cruzi* -negative 16 | Negative | 0.05 | 0.29 | 0.46 | 0.50 | 0.50 |
| *Trypanosoma cruzi* -negative 17 | Negative | 0.17 | 0.49 | 0.46 | 0.52 | 0.67 |
| *Trypanosoma cruzi* -negative 18 | Negative | 0.16 | 0.66 | 0.46 | 0.32 | 1.42 |
| *Trypanosoma cruzi* -negative 19 | Negative | 0.09 | 0.40 | 0.48 | 0.32 | 0.53 |
| *Trypanosoma cruzi* -negative 20 | Negative | 0.07 | 0.53 | 0.51 | 0.60 | 0.65 |
| *Trypanosoma cruzi* -negative 21 | Negative | 0.16 | 0.40 | 0.62 | 0.42 | 0.49 |
| *Trypanosoma cruzi* -negative 22 | Negative | 0.25 | 0.49 | 0.58 | 0.56 | 1.52 |
| *Trypanosoma cruzi* -negative 23 | Negative | 0.33 | 0.45 | 0.96 | 0.44 | 0.40 |
| *Trypanosoma cruzi* -negative 24 | Negative | 0.14 | 0.59 | 0.78 | 0.46 | 0.60 |
| *Trypanosoma cruzi* -negative 25 | Negative | 0.83 | 0.47 | 0.60 | 0.28 | 1.03 |
| *Trypanosoma cruzi* -negative 26 | Negative | 0.31 | 0.47 | 0.72 | 0.26 | 0.82 |
| *Trypanosoma cruzi* -negative 27 | Negative | 0.16 | 0.39 | 0.49 | 0.34 | 0.56 |
| *Trypanosoma cruzi* -negative 28 | Negative | 0.72 | 0.48 | 0.58 | 0.31 | 1.07 |
| *Trypanosoma cruzi* -negative 29 | Negative | 0.12 | 0.85 | 0.63 | 0.35 | 1.04 |
| *Trypanosoma cruzi* -negative 30 | Negative | 0.69 | 0.59 | 0.56 | 0.58 | 0.61 |
| *Trypanosoma cruzi* -negative 31 | Negative | 0.58 | 0.42 | 0.56 | 0.69 | 0.73 |
| *Trypanosoma cruzi* -negative 32 | Negative | 0.34 | 0.72 | 0.46 | 0.59 | 0.65 |
| *Trypanosoma cruzi* -negative 33 | Negative | 0.36 | 0.27 | 0.62 | 0.46 | 0.37 |
| *Trypanosoma cruzi* -negative 34 | Negative | 0.23 | 0.73 | 0.58 | 0.69 | 0.82 |
| *Trypanosoma cruzi* -negative 35 | Negative | 0.24 | 0.52 | 0.51 | 0.71 | 0.90 |
| *Trypanosoma cruzi* -negative 36 | Negative | 0.30 | 0.50 | 0.40 | 0.49 | 0.65 |
| *Trypanosoma cruzi* -negative 37 | Negative | 0.12 | 0.54 | 0.49 | 0.51 | 0.59 |
| *Trypanosoma cruzi* -negative 38 | Negative | 0.26 | 0.63 | 0.42 | 0.42 | 0.69 |
| *Trypanosoma cruzi* -positive 1 | Indeterminate form | 2.20 | 8.57 | 1.94 | 1.67 | 0.84 |
| *Trypanosoma cruzi* -positive 2 | Indeterminate form | 2.46 | 14.80 | 2.42 | 0.83 | 0.47 |
| *Trypanosoma cruzi* -positive 3 | Indeterminate form | 2.20 | 14.12 | 0.92 | 1.58 | 0.43 |
| *Trypanosoma cruzi* -positive 4 | Indeterminate form | 2.68 | 16.34 | 1.01 | 4.70 | 0.81 |
| *Trypanosoma cruzi* -positive 5 | Indeterminate form | 2.67 | 14.56 | 6.22 | 0.95 | 0.72 |
| *Trypanosoma cruzi* -positive 6 | Indeterminate form | 1.93 | 1.16 | 2.17 | 1.54 | 0.79 |
| *Trypanosoma cruzi* -positive 7 | Indeterminate form | 2.86 | 13.88 | 1.28 | 1.31 | 0.45 |
| *Trypanosoma cruzi* -positive 8 | Indeterminate form | 1.91 | 8.95 | 2.39 | 2.28 | 1.38 |
| *Trypanosoma cruzi* -positive 9 | Indeterminate form | 1.61 | 9.49 | 0.63 | 0.42 | 0.34 |
| *Trypanosoma cruzi* -positive 10 | Indeterminate form | 1.98 | 6.43 | 0.86 | 0.64 | 0.64 |
| *Trypanosoma cruzi* -positive 11 | Indeterminate form | 1.81 | 11.52 | 0.49 | 0.72 | 0.98 |
| *Trypanosoma cruzi* -positive 12 | Indeterminate form | 1.31 | 9.76 | 1.01 | 0.50 | 0.91 |
| *Trypanosoma cruzi* -positive 13 | Indeterminate form | 2.31 | 8.55 | 0.70 | 1.02 | 0.81 |
| *Trypanosoma cruzi* -positive 14 | Indeterminate form | 1.95 | 3.52 | 1.68 | 2.18 | 0.76 |
| *Trypanosoma cruzi* -positive 15 | Indeterminate form | 2.07 | 3.09 | 1.13 | 3.02 | 0.91 |
| *Trypanosoma cruzi* -positive 16 | Indeterminate form | 4.97 | 3.33 | 1.21 | 1.25 | 0.69 |
| *Trypanosoma cruzi* -positive 17 | Indeterminate form | 1.86 | 3.78 | 0.86 | 0.44 | 1.09 |
| *Trypanosoma cruzi* -positive 18 | Indeterminate form | 2.85 | 1.77 | 0.58 | 0.75 | 0.79 |
| *Trypanosoma cruzi* -positive 19 | Indeterminate form | 2.12 | 0.93 | 0.68 | 1.44 | 1.36 |
| *Trypanosoma cruzi* -positive 20 | Indeterminate form | 4.92 | 2.47 | 0.78 | 0.49 | 1.79 |
| *Trypanosoma cruzi* -positive 21 | Indeterminate form | 2.13 | 0.54 | 0.58 | 0.67 | 0.93 |
| *Trypanosoma cruzi* -positive 22 | Indeterminate form | 3.45 | 1.75 | 0.94 | 0.39 | 0.93 |
| *Trypanosoma cruzi* -positive 23 | Indeterminate form | 3.19 | 2.18 | 1.26 | 0.73 | 0.91 |
| *Trypanosoma cruzi* -positive 24 | Indeterminate form | 3.56 | 1.04 | 1.83 | 1.39 | 0.82 |
| *Trypanosoma cruzi* -positive 25 | Severe cardiac form | 3.25 | 1.05 | 0.83 | 1.09 | 1.20 |
| *Trypanosoma cruzi* -positive 26 | Severe cardiac form | 1.49 | 1.19 | 0.56 | 0.89 | 0.85 |
| *Trypanosoma cruzi* -positive 27 | Severe cardiac form | 3.02 | 5.58 | 0.76 | 0.87 | 1.08 |
| *Trypanosoma cruzi* -positive 28 | Severe cardiac form | 1.44 | 2.85 | 0.63 | 0.68 | 1.21 |
| *Trypanosoma cruzi* -positive 29 | Severe cardiac form | 2.46 | 2.07 | 0.78 | 1.31 | 1.38 |

| | | | | | | |
|---|---|---|---|---|---|---|
| *Trypanosoma cruzi* -positive 30 | Severe cardiac form | 2.39 | 3.34 | 3.27 | 0.64 | 1.46 |
| *Trypanosoma cruzi* -positive 31 | Severe cardiac form | 2.55 | 10.92 | 0.96 | 1.28 | 0.57 |
| *Trypanosoma cruzi* -positive 32 | Severe cardiac form | 2.58 | 9.20 | 1.04 | 0.48 | 0.61 |
| *Trypanosoma cruzi* -positive 33 | Severe cardiac form | 3.88 | 19.89 | 3.08 | 0.84 | 1.13 |
| *Trypanosoma cruzi* -positive 34 | Severe cardiac form | 2.01 | 4.81 | 1.18 | 0.34 | 0.84 |
| *Trypanosoma cruzi* -positive 35 | Severe cardiac form | 3.02 | 13.26 | 0.88 | 0.53 | 0.63 |
| *Trypanosoma cruzi* -positive 36 | Severe cardiac form | 1.60 | 3.34 | 0.67 | 0.31 | 1.41 |
| *Trypanosoma cruzi* -positive 37 | Severe cardiac form | 3.87 | 21.85 | 0.85 | 1.06 | 1.19 |
| *Trypanosoma cruzi* -positive 38 | Severe cardiac form | 2.99 | 18.40 | 1.91 | 1.07 | 1.61 |
| *Trypanosoma cruzi* -positive 39 | Severe cardiac form | 2.39 | 8.59 | 0.82 | 1.13 | 0.46 |
| *Trypanosoma cruzi* -positive 40 | Mild cardiac form | 3.00 | 17.01 | 1.40 | 0.55 | 1.13 |
| *Trypanosoma cruzi* -positive 41 | Mild cardiac form | 2.65 | 6.97 | 2.99 | 0.52 | 0.57 |
| *Trypanosoma cruzi* -positive 42 | Mild cardiac form | 2.97 | 18.29 | 1.55 | 0.75 | 0.67 |
| *Trypanosoma cruzi* -positive 43 | Mild cardiac form | 2.88 | 7.29 | 0.97 | 1.97 | 1.51 |
| *Trypanosoma cruzi* -positive 44 | Mild cardiac form | 2.90 | 20.75 | 1.72 | 1.00 | 1.63 |
| *Trypanosoma cruzi* -positive 45 | Mild cardiac form | 3.20 | 20.96 | 1.02 | 0.43 | 1.85 |
| *Trypanosoma cruzi* -positive 46 | Mild cardiac form | 3.52 | 18.14 | 0.86 | 1.55 | 1.98 |
| *Trypanosoma cruzi* -positive 47 | Mild cardiac form | 1.98 | 4.78 | 0.61 | 0.39 | 1.02 |
| *Trypanosoma cruzi* -positive 48 | Mild cardiac form | 3.83 | 19.69 | 0.71 | 1.21 | 0.55 |
| *Trypanosoma cruzi* -positive 49 | Mild cardiac form | 3.40 | 14.67 | 0.57 | 3.14 | 0.70 |
| *Trypanosoma cruzi* -positive 50 | Mild cardiac form | 2.42 | 4.41 | 1.34 | 1.61 | 0.69 |
| *Trypanosoma cruzi* -positive 51 | Mild cardiac form | 3.37 | 16.57 | 4.97 | 2.79 | 1.95 |
| *Trypanosoma cruzi* -positive 52 | Mild cardiac form | 3.02 | 10.39 | 7.66 | 0.52 | 1.10 |
| *Trypanosoma cruzi* -positive 53 | Mild cardiac form | 3.77 | 20.55 | 1.15 | 5.68 | 3.36 |
| *Trypanosoma cruzi* -positive 54 | Mild cardiac form | 1.94 | 8.08 | 0.85 | 1.58 | 0.99 |
| *Trypanosoma cruzi* -positive 55 | Mild cardiac form | 3.12 | 9.41 | 0.89 | 1.68 | 1.61 |
| *Trypanosoma cruzi* -positive 56 | Mild cardiac form | 1.54 | 1.75 | 3.83 | 1.17 | 0.64 |
| *Trypanosoma cruzi* -positive 57 | Mild cardiac form | 3.03 | 14.77 | 1.17 | 1.99 | 0.56 |
| *Trypanosoma cruzi* -positive 58 | Mild cardiac form | 3.16 | 15.55 | 1.03 | 1.61 | 0.68 |
| *Trypanosoma cruzi* -positive 59 | Mild cardiac form | 3.68 | 11.95 | 3.89 | 4.15 | 0.55 |
